# Understanding Defects in Perovskite Solar Cells through Computation: Current Knowledge and Future Challenge

**DOI:** 10.1002/advs.202305799

**Published:** 2024-03-19

**Authors:** Zhendong Guo, Man Yuan, Gaoyuan Chen, Feng Liu, Ruifeng Lu, Wan‐Jian Yin

**Affiliations:** ^1^ Department of Applied Physics Nanjing University of Science and Technology Nanjing 210094 China; ^2^ College of Energy Soochow Institute for Energy and Materials InnovationS (SIEMIS) and Jiangsu Provincial Key Laboratory for Advanced Carbon Materials and Wearable Energy Technologies Soochow University Suzhou 215006 China; ^3^ Jiangsu Key Laboratory of Micro and Nano Heat Fluid Flow Technology and Energy Application School of Physical Science and Technology Suzhou University of Science and Technology Suzhou 215009 China; ^4^ College of Energy Soochow Institute for Energy and Materials InnovationS (SIEMIS) Soochow University Suzhou 215006 China; ^5^ Light Industry Institute of Electrochemical Power Sources Soochow University Suzhou 215006 China

**Keywords:** defects, machine learning, nonradiative recombination, perovskite, phase degradation

## Abstract

Lead halide perovskites with superior optoelectrical properties are emerging as a class of excellent materials for applications in solar cells and light‐emitting devices. However, perovskite films often exhibit abundant intrinsic defects, which can limit the efficiency of perovskite‐based optoelectronic devices by acting as carrier recombination centers. Thus, an understanding of defect chemistry in lead halide perovskites assumes a prominent role in further advancing the exploitation of perovskites, which, to a large extent, is performed by relying on first‐principles calculations. However, the complex defect structure, strong anharmonicity, and soft lattice of lead halide perovskites pose challenges to defect studies. In this perspective, on the basis of briefly reviewing the current knowledge concerning computational studies on defects, this work concentrates on addressing the unsolved problems and proposing possible research directions in future. This perspective particularly emphasizes the indispensability of developing advanced approaches for deeply understanding the nature of defects and conducting data‐driven defect research for designing reasonable strategies to further improve the performance of perovskite applications. Finally, this work highlights that theoretical studies should pay more attention to establishing close and clear links with experimental investigations to provide useful insights to the scientific and industrial communities.

## Graphical abstract

A deep understanding of defect chemistry in lead‐halide perovskites is vitally important. On the basis of reviewing current knowledge concerning computational studies about defects, controversial problems concerning the role of defects on carrier recombination and phase degradation, as well as possible research directions are proposed, with emphasizing the construction of close links between theoretical and experimental investigations via machine learning.

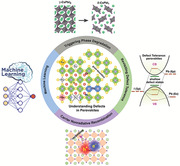

## 1 Introduction

The efficiency of solar cells based on lead halide perovskites (LHPs) has improved unprecedentedly during the past decade. The power conversion efficiency (PCE) has increased rapidly from 3.8% (2009)^[^ ^1^ ^]^ to the currently certified 26.1% (2023),^[^ ^2^ ^]^ demonstrating the potential of LHPs to compete with established thin‐film technologies, including polycrystalline silicon (*p*‐Si), cadmium telluride (CdTe), and copper indium gallium (di)selenide (CIGS) solar cells.^[^ ^3^ ^]^ The outstanding performance of perovskite solar cells (PSCs) significantly benefits from the superior photophysical properties of LHPs, such as high light‐absorption coefficient, suitable band gap, small exciton binding energy, long charge diffusion path, and low carrier recombination rate.

**Figure 1:**
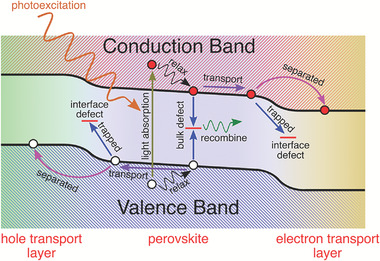
Schematic illustration of the dynamic behaviors of photoactivated carriers in PSCs, highlighting the recombination of carriers induced by the bulk and interfacial defects (blue arrows) possessing gap states.

Despite the remarkable success of PSCs, their wide commercial application remains hindered by obstacles. First, considering the maximum theoretical efficiency of ≈33% for a single‐junction solar cell based on the Shockley–Queisser analysis,^[^ ^4^ ^]^ numerous areas for further improvement of the PCE of PSCs remain. On the material level, perovskite films often feature abundant intrinsic defects, such as antisites, interstitials, and vacancies, as well as impurities and dangling bonds at the grain boundaries (GBs) and surfaces, which may result in gap states that significantly contribute to the nonradiative recombination of photo‐activated carriers (cf. **Figure** **1**). On the device level, such undesired recombination inevitably increases the open‐circuit voltage deficit, thus acting as an important roadblock toward achieving the theoretical efficiency limit. Second, the weak long‐term stability of LHPs means that the PSCs cannot satisfy the basic requirement of commercial applications with working lifetimes exceeding 20 years. Besides the thermal decomposition of volatile organic cations, the degradation of LHPs from the moisture, oxygen, and light illumination also severely shorten the lifetime of PSCs, and the presence of defects supposedly mediates this degradation.^[^ ^5^, ^6^, ^7^ ^]^ In addition, the undesired phase transition of FAPbI_3_ and CsPbI_3_ from perovskite to non‐perovskite phases rapidly converts the superior light‐absorption layer into the optical dead‐layer within several days,^[^ ^8^, ^9^ ^]^ thereby resulting in substantially lowered PCE of PSCs, which is thought to be triggered or accelerated by defects.^[^ ^10^, ^11^ ^]^ Finally, poor reproducibility and current–voltage hysteresis, which are linked to defect formation and migration, remain an unsolved problem.^[^ ^12^, ^13^ ^]^ To sum up, perovskite defects are responsible for the majority of bottlenecks that urgently need to be overcome for the final commercialization of PSCs. Therefore, fundamentally understanding defect chemistry in perovskites becomes a critically important issue, which provides researchers not only deeper insights concerning LHPs but also promising directions to attain the final solution to the aforementioned obstacles.

Extensive studies have been conducted aiming to clarify the defect chemistry in perovskites. Among them, theoretical calculations based on first‐principles play an important role because they investigate and understand defect properties at the atomic level. Owing to the contribution from theoretical studies, some basic consensus regarding defect chemistry in perovskites has been attained, including its defect tolerance and fast defect movement. Despite previous efforts, the nature and activity of defects in LHPs and the underlying mechanism of how they impact the performance of PSCs have remained elusive. In this work, we attempt to raise the open questions and existing controversy about defect chemistry in LHPs, aiming to stimulate more attention into this field and promote the improvement of PSCs.

## 2 What We Can and Cannot Do in Defect Calculations

As shown in Figure 1, defects play crucial roles in all processes of photo‐excited carriers, such as relaxation, recombination, transportation, and separation. What can be done for defect calculations?

### 2.1 Formation Energy

Formation energy can be calculated using the following equation:^[^ ^14^, ^15^, ^16^ ^]^

(1) $$E^{f}\;\left[X^{q}\right]=E_{\mathrm{tot}}\;\left[X^{q}\right]-E_{\mathrm{tot}}\left[\mathrm{bulk}\right]-\sum_{i}n_{i}\mu_{i}+q\left(E_{F}+\varepsilon_{v}\right)+E_{\mathrm{corr}}$$

where *E*_tot_[*X^q^*] is the total energy of the supercell with a defect *X* in the charge state *q*, *E*_tot_[bulk] is the total energy of the perfect supercell, *n_i_* is the number of added (*n_i_* > 0) or subtracted (*n_i_* < 0) atoms of the species *i* needed to create the defect *X*, and µ_*i*_ is the respective chemical potential. *E_F_* represents the Fermi level with respect to the position of VBM (ε_*v*_). Finally, *E*_corr_ is a correction term that accounts for finite **k**‐point sampling in the case of shallow impurities or for elastic and/or electrostatic interactions between supercells. Why do we care so much about the formation energy (*E^f^* [*X^q^*]) of one defect? That is because it can be directly used to quantitatively assess how easy one defect forms in the host. In short, the higher the formation energy is, the more difficultly the defect appears, implying the lower defect concentration.

**Figure 2:**
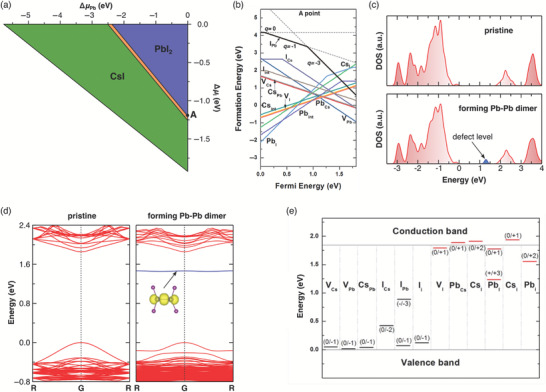
a) Thermodynamic stability diagram of γ‐CsPbI_3_ as a function of the relative chemical potentials of lead (Δµ_Pb_) and iodine (Δµ_I_). The narrow orange‐shaded area shows the region where γ‐CsPbI_3_ is thermodynamically stable. Point A marks the representative I‐poor (Pb‐rich) condition, with Δ µ_Cs_= −2.15 eV, Δ µ_Pb_= 0.00 eV and Δ µ_I_= −1.20 eV. b) Calculated formation energies of intrinsic point defects in γ‐CsPbI_3_ with chemical potentials at A point in (a). c,d) Comparison of total density of states (DOS) (c) and band structures (d) for pristine and defective γ‐CsPbI_3_ with the formation of Pb‐Pb dimer. e) Calculated charge transition energy levels of intrinsic point defects in γ‐CsPbI_3_. b,e) reproduced with permission.^[^ ^17^ ^]^ Copyright 2017, American Chemical Society.

**Figure 3:**
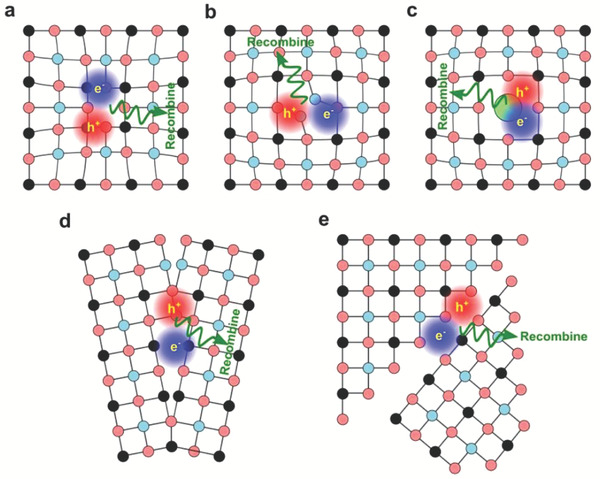
Schematic illustration of point defects (a) vacancy; b) interstitial; c) substitution), d) grain and e) interphase boundaries similar to a perovskite crystal lattice with light blue, black, and light red dots representing the A‐, B‐, and X‐site ions, respectively.

It can be seen from Equation (1) that *E^f^* [*X^q^*] heavily depends on the values of µ_*i*_ and *E_F_*, both of which can be tuned in experiment. Very often the chemical potential µ_*i*_ is referenced to the atomic energy *E_i_* of pure elemental solid/gas of *i*, that is, µ_*i*_ = *E_i_*  + Δµ_*i*_. Under the thermodynamic equilibrium growth conditions, a series of limitations has been set on µ_*i*_, making Δµ_*i*_ confined in a finite range. Let us take CsPbI_3_ as an example to illustrate this. First, to avoid the formation of elemental solid/gas, µ_*i*_ (*i* = Cs, Pb and I) should be smaller than *E_i_*, namely Δµ_*i*_ < 0. Second, the formation of competing compounds (like CsI and PbI_2_) should also be prevented, which needs µ_Cs_ + µ_I_ < *E*_CsI_ and $\mu_{\mathrm{Pb}}+2\mu_{I}<E_{{\mathrm{PbI}}_{2}}$. When considering µ_Cs_ = *E*_Cs_  + Δµ_Cs_, µ_Pb_ = *E*_Pb_  + Δµ_Pb_ and µ_I_ = *E*_I_  + Δµ_I_, the aforementioned two inequality constraints can be reformulated as:

(2) $$\Delta \mu_{\mathrm{Cs}}+\Delta \mu_{I}<E_{\mathrm{CsI}}-E_{\mathrm{Cs}}-\;E_{I}=\;\Delta H_{f}\left(\mathrm{CsI}\right)$$

(3) $$\Delta \mu_{\mathrm{Pb}}+2\Delta \mu_{I}<E_{{\mathrm{PbI}}_{2}}-E_{\mathrm{Pb}}-2\;E_{I}=\;\Delta H_{f}\left({\mathrm{PbI}}_{2}\right)$$

where Δ*H_f_* (CsI) and Δ*H_f_* (PbI_2_) represent the formation enthalpy of CsI and PbI_2_, respectively. Finally, to maintain the stable CsPbI_3_ compound, the chemical potentials µ_Cs_, µ_Pb_ and µ_I_ should also satisfy: $\mu_{\mathrm{Cs}}+\mu_{\mathrm{Pb}}+3\;\mu_{I}=E_{{\mathrm{CsPbI}}_{3}}\;$, which can also be reformulated as:

(4) $$\begin{array}{ccc} \Delta \mu_{\mathrm{Cs}} & + & \Delta \mu_{\mathrm{Pb}}+3\Delta \;\mu_{I}=E_{{\mathrm{CsPbI}}_{3}}\;-E_{\mathrm{Cs}}-E_{\mathrm{Pb}}-3\;E_{I}\\ & & =\;\Delta H_{f}\left({\mathrm{CsPbI}}_{3}\right) \end{array}$$

where Δ*H_f_* (CsPbI_3_) represents the formation enthalpy of CsPbI_3_. The relative chemical potentials of Pb (Δµ_Pb_) and I (Δµ_I_) satisfying Equations ((2), (3), (4)) are shown within the narrow orange region in **Figure** **2**a. Hence, Δµ_*i*_ close to zero corresponds to the *i*‐rich growth condition, which indicates the high concentration/ratio of the element *i* in the precursors for the growth of perovskites. Accordingly, the situation of low Δµ_*i*_ can be interpreted as the *i*‐poor condition. For defects of *i*‐vacancy (cf. **Figure** **3**a), *n_i_* < 0 in Equation (1), the higher Δµ_*i*_ (the *i*‐rich condition) will enhance the defect formation energy, and then lower the defect concentration. For defects of *i*‐interstitial (cf. Figure 3b), *n_i_* > 0 in Equation (1), the lower Δµ_*i*_ (the *i*‐poor condition) tends to enhance the defect formation energy and decrease the defect ratio. For defects of *i* substituted by *j* (cf. Figure 3c), *n_i_* < 0 and *n_j_* > 0 in Equation (1), the higher Δµ_*i*_ (the *i*‐rich condition) and the lower Δµ_*j*_ (the *j*‐poor condition) will increase the defect formation energy and inhibit the appearance of defects.

Besides the chemical potentials of elements, the position of Fermi level (*E_F_*), varying in the gap, also significantly influences the value of *E^f^* [*X^q^*], which shows the linear dependence on *E_F_*, with the slope of *q*. Usually, for one specific defect, its stable charge state *q* is not fixed, which changes with the varying *E_F_*. In order to determine the stable charge state for the defect *X*, we need to consider multiple charge states (e.g. *q* = +4, +3, +2, +1, 0, −1, −2, −3, and −4), fully relax the configurations of *X^q^* under different charge states, calculate and plot a series of lines of *E^f^* [*X^q^*] versus *E_F_* with different slopes (*q*) according to Equation (1), which are illustrated by the gray dashed lines for the defect of I_Pb_ (one Pb atom replaced by one I atom) in Figure 2b. It can be noticed that the lowest formation energy for I_Pb_ corresponds to different lines and charge states when *E_F_* located in different regions of the gap. We can connect these segments corresponding to the lowest formation energy marked by the solid black curve in Figure 2b, which demonstrates the evolution of the formation energy and stable charge state (from 0 to −1 and then −3) for I_Pb_ as *E_F_* varies from VBM to CBM. Because the other lines for *q* = +4, +3, +2, +1, −2, and −4 always indicate higher formation energy than that of the solid black curve, these lines were not shown in Figure 2b. Generally, theoretical researchers will just show the final curve corresponding to the lowest formation energy of one defect rather than a series of lines with different slopes (*q*) in their work, like the cases for other defects in Figure 2b. It should be noted that the choice of different chemical potentials will not change the shape of the lowest formation energy curve as well as the stable charge state, but just entirely pushes up or down this curve. In summary, in order to solve the formation energy of one defect, we need to first choose appropriate chemical potentials, which should reflect the growth conditions of the host material. After that, we need to determine the lowest formation energy curve by calculating a series of formation energy lines as a function of *E_F_*.

### 2.2 Single‐Electron Level

Defects can be considered as a perturbation to the periodic lattice (cf. Figure 3); therefore, the defect level can be viewed as the bound state in a screening central perturbing the crystal field, where the ionization energy ε is given by:^[^ ^18^ ^]^

(5) $$\varepsilon \;=\frac{e^{4}m^{*}}{32\pi^{2}\varepsilon_{0}^{2}\varepsilon^{2}\hbar^{2}}\;$$

where *e* represents the elementary charge, m* is the effective mass of carriers, ε_0_ is the vacuum permittivity, ε is the relative permittivity, and ℏ is the reduced Planck constant. Equation (5), which is derived from a spheric central field, indicates that small m* and large ε may lead to shallow defect levels.

In most cases, electronic structure calculations in a large supercell with/without defects are performed by DFT calculations, and the defect levels can be directly identified by comparing the density of states (cf. Figure 2c) and band structures (cf. Figure 2d) of model systems with and without defects. Despite their problematic principles, such direct approaches are widely used in the field as a rough estimate to judge whether a defect existing in the bulk, surface, GBs, or interface is benign or detrimental.

### 2.3 Thermodynamic Transition Level

One of the problems associated with single‐electron level is that it is based on the one‐electron theory in ground‐state calculations, failing to account for atomic displacement before and after the capture of electrons or holes. In halide perovskites with soft lattice, such atomic displacement can be large.^[^ ^19^, ^20^ ^]^ In order to perform relatively rigorous studies^[^ ^14^, ^21^, ^22^ ^]^ to check whether a defect is shallow or deep, the corresponding charge transition level ε(*q*_1_/*q*_2_) should be calculated (cf. Figure 2e), which is defined as the Fermi‐level position at which the formation energies of defect $X^{q_{1}}$ and $X^{q_{2}}$ are equal:

(6) $$\varepsilon \left(q_{1}/q_{2}\right)=\frac{E^{f}\left[X^{q_{1}};\;E_{F}=\;0\right]-E^{f}\left[X^{q_{2}};\;E_{F}=\;0\right]}{q_{2}-q_{1}}$$

It should be noted that the charge transition levels are independent of the choice of chemical potentials of elements. Transition levels have experimental significance that can be observed in experiments such as deep‐level transient spectroscopy (DLTS) and corresponds to thermal ionization energies. For investigating transition levels of defects in LHPs, more attention and efforts are still required, as recent work has reported that the semilocal and hybrid functionals give an opposite judgement about the electrical properties of iodine vacancy (V_I_), one predicting the deep transition level for V_I_ and the other implying no defect level in the gap.^[^ ^23^ ^]^ Usually, the semilocal and hybrid functionals will provide the consistent judgement about one defect, and the application of hybrid functional can better repeat the experimental gap which is generally underestimated by semilocal functionals. But for LHPs, the semilocal functionals yield more or less the right band gap due to an error cancellation, with neglecting the influence of spin‐orbit coupling (SOC) effect.^[^ ^24^ ^]^ Because of the high‐efficiency and the right prediction of bandgap, semilocal functionals represented by the PBE functional are widely adopted by researchers to investigate defects in LHPs. But, in principle, the hybrid functionals in conjunction with SOC should be the optimal choice, as hybrid functionals are thought to describe structures more accurately and the SOC effect in LHPs should be considerable due to the heavy Pb atoms. In this context, it is rather necessary to carefully re‐investigate defect properties in LHPs, being not limited to charge transition levels, by adopting the aforementioned combination of hybrid functionals and SOC.

### 2.4 Configuration Coordination Diagram

**Figure 4:**
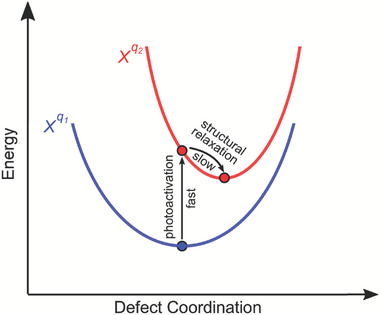
Schematic representation of the photoactivation and structural relaxation processes for defect *X* with the charge state transferring from *q_1_* to *q_2_*.

In Equation (1), all energy terms for charged defects are calculated in their ground state, meaning that the defects have relaxed to their ground states after capturing an electron or hole. This assumption is true for electrical cases such as the measurement of DLTS, in which the timescale for Fermi level scanning is considerably larger than that of atomic relaxation process; thus, the investigation of electrically active defects only aims at their ground states. Meanwhile, the optical process associated with the injection of charges for defects occurs extremely fast and actually corresponds to a vertical transition process, with no time left for thermodynamically structural relaxation. For describing this fast optical process, the aforementioned single electron‐level rather than the charge transition level should be considered. A configuration coordination diagram is necessary to understand the photoexcitation process as shown in **Figure** **4**.

### 2.5 Defect Concentration

Considering all the crucial defects in a system, the defect concentrations can be obtained using the following equation:

(7) $$N\;\left[X^{q}\right]=N_{\mathrm{site}}\;\cdot \exp\left[\frac{-E^{f}\left[X^{q}\right]}{k_{B}T}\right]$$

where *N*_site_ is the number of available sites per volume that may be occupied by the defect *X*, *E^f^* [*X^q^*] is the formation energy of the defect *X* with the charge state *q*, *k_B_* is the Boltzmann constant, and *T* is the growth temperature. Let us also take γ‐CsPbI_3_ as an example to illustrate how we get the equilibrium defect concentrations and Fermi level positions. Here, one precondition is that we have obtained the lowest formation energy curves for all intrinsic point defects in γ‐CsPbI_3_, like results shown in Figure 2b. First, we need to calculate the total charges of the whole system by considering the contributions of all studied defects (∑*q* · *N*[*X^q^*]), according to Equation (7) with putting *E_F_* at VBM and CBM, respectively. It can be seen from Figure 2b that the positively charged defects (e.g. Cs_int_, V_I_, Cs_I_, Pb_Cs_, Pb_int_, and Pb_I_) have relatively low formation energies and high concentrations when *E_F_* located in the lower part of the gap, while the negatively charged defects (e.g. I_Pb_, I_Cs_, I_int_, V_Cs_, Cs_Pb_, and V_Pb_) possess relatively low formation energies and high concentrations when *E_F_* located in the upper part of the gap. So, it can be known that the whole system contains the positive and negative total charge when *E_F_* at VBM and CBM, respectively. Under the charge neutralization condition for the whole system, the equilibrium Fermi level should lie between VBM and CBM. We set *E*_*F*1_ = VBM, *E*_*F*2_ = CBM and *E*_*F*0_ = (*E*_*F*1_+*E*_*F*2_)/2, and calculate the total charge for the whole system with *E_F_* = *E*_*F*0_ . If the total charge is positive, we update *E*_*F*1_ = *E*_*F*0_ and reset *E*_*F*0_ = (*E*_*F*1_+*E*_*F*2_)/2. Otherwise, we update *E*_*F*2_ = *E*_*F*0_ and then *E*_*F*0_ = (*E*_*F*1_+*E*_*F*2_)/2. This updating mechanism ensures that the equilibrium Fermi level always lies between *E*_*F*1_ and *E*_*F*2_. After that, we recalculate the total charge of the whole system with *E_F_* = *E*_*F*0_ . By iteratively repeating this process, the total charge will rapidly converge to 0, with *E*_*F*0_ under this occasion corresponding to the equilibrium Fermi level. And then, the concentration of every defect can be calculated by adopting the equilibrium Fermi level according to Equation (7). Certain codes are available to help in this calculation, for example, the DASP package.^[^ ^25^ ^]^

### 2.6 Recombination Rate

**Figure 5:**
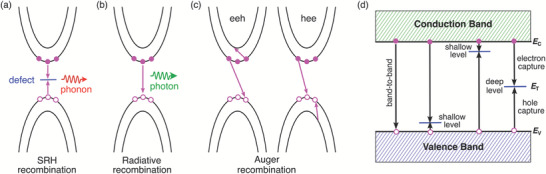
Schematic illustration of different recombination processes: a) Shockley–Read–Hall (SRH) recombination, b) radiative recombination, and c) Auger recombination including electron–electron–hole (eeh) and hole–hole–electron (hhe) processes. The pink solid and open spheres denote electrons and holes, respectively. d) Two steps occurring in the recombination of an electron‐hole pair mediated by the defect level in the SRH theory.

The recombination of charge carriers in a semiconductor can be summarized through the following simplified rate equation:^[^ ^26^, ^27^ ^]^

(8) $$-\;\frac{dn}{dt}=k_{1}\;n+k_{2}n^{2}+k_{3}n^{3}$$

where *n* is the electron charge carrier density (assuming electron density equals hole density to facilitate qualitative discussion in Equation (8)), *k*_1_ is the first‐order Shockley−Read−Hall (SRH) trapping (nonradiative) rate constant, *k*_2_ is the second‐order band‐to‐band (radiative) recombination rate constant, and *k*_3_ is the third‐order Auger (nonradiative) recombination rate constant. The aforementioned three recombination processes (SRH, radiative and Auger recombination) are illustrated schematically in **Figure** **5**a–c. Auger recombination is generally much weaker than other recombination channels at normal solar cell operating conditions (under 1 sun illumination with the carrier concentration of 10^15^–10^16^ cm^−3^).^[^ ^28^ ^]^ In perovskites, it is widely accepted that the radiative recombination is strongly suppressed by the Rashba effect,^[^ ^29^ ^]^ which explains the long carrier lifetime observed in experiments. In this context, SRH recombination mediated by defect levels (cf. Figure 5a) mainly contributes to the loss of photo‐activated carriers in perovskites, which process can be well described by the SRH model proposed in 1952.^[^ ^30^, ^31^ ^]^ In SRH model, the recombination of an electron‐hole pair needs two steps corresponding to the hopping of an electron from CBM (*E*_C_) to the trap level (*E*_T_) and the hopping of a hole from VBM (*E*_V_) to *E*_T_ (cf. Figure 5d), which are known as electron and hole capture, respectively. In SRH theory, the electron (*C_n_*) and hole (*C_p_*) capture rate both heavily depend on the position of *E*_T_, with $C_{n}\propto \exp\left(-\frac{E_{C}-E_{T}}{k_{B}T}\right)$ and $C_{p}\propto \exp\left(-\frac{E_{T}-E_{V}}{k_{B}T}\right)$. So, we can know that when *E*_T_ is very close to CBM, *C_p_* will become very small, while *C_n_* becomes limited as *E*_T_ close to VBM. Generally, the defect level close to the band edges (VBM or CBM) with a gap less than 0.2 eV is called the shallow level and thought to be benign, as its acceleration effect for carrier recombination is limited. But when *E*_T_ is located at the central region of the gap and the defect level is called the deep level, *C_n_* and *C_p_* will become considerable simultaneously, which defect level is thought to be detrimental and significantly promote the carrier recombination. Therefore, to attain the high efficiency of solar cells, any defect generating deep levels should be avoided. Here, we can know that the calculation of transition level or single‐electron level may provide a qualitative measurement of whether a defect is benign (shallow) or detrimental (deep), according to SRH theory. In order to obtain a quantitative description of carrier relaxation, trap, and recombination, the contribution of ionic movement should be considered.^[^ ^32^, ^33^, ^34^ ^]^ The core quantity to accurately judge the benign or detrimental nature of a defect is the associated nonradiative recombination rate, which can generally be calculated by two types of methods: one is based on the calculation of electron–phonon coupling,^[^ ^33^, ^35^, ^36^, ^37^, ^38^ ^]^ and the other is based on non‐adiabatic molecular dynamics simulations.^[^ ^39^, ^40^, ^41^, ^42^ ^]^

Recent developments in the methodologies of carrier dynamics calculations have achieved considerable progress in quantitatively describing the carrier relaxation, trap, and recombination in LHPs. However, a quantitative comparison of these approaches with experiments is questionable, with certain theoretical results scattered in several orders of magnitude. Quantitative consistency with experimental measurements has been claimed in certain cases, but such claims should be carefully made because experimental situations could be much more complicated and possibly contain numerous defects, GBs, and surfaces/interfaces. Moreover, such types of calculations are extremely time‐consuming and usually adopt small supercells with defect concentrations (10^20^ cm^−3^ when one defect is in a 100‐atom cell) that are much larger than that of experimental reality (10^12^–10^15^ cm^−3^). How the calculated results scale with the defect concentration is an unavoidable problem that needs to be addressed for practical comparison between theoretical calculations and experimental measurements.^[^ ^43^ ^]^

Moreover, current static and dynamic calculations are mostly focused on single‐point defect/defect pairs. To meet the device level, computation should be advanced to the overall point defects, GBs, interface, and interphase. Certain challenges to this approach are the computational cost and real atomistic structures of the defects that require experimental observations.

Until now, researchers have spent a huge amount of effort to clarify how defects influence the carrier recombination. But we should realize that the injection of carriers under the light illumination can also react upon the dynamical and electrical behaviors of defects, which also deserve attention. Tong et al. have investigated how hole injection accelerates both ion migration and nonradiative recombination in MAPbI_3_.^[^ ^44^ ^]^ Wang et al. reported carriers generated by light illumination promote ion‐diffusion‐assisted transitions of detrimental defects in MAPbI_3_.^[^ ^45^ ^]^ Although some attempts in this direction have been performed, the behaviors of defects under the continuous injections of carriers are still unclear, and call for further studies. Maybe those investigations are quite important for the development of PSCs, as plenty of carriers always exist and influence intrinsic defects in PSCs under the working conditions, which were consciously neglected in previous theoretical studies.

## 3 Revisiting Defect Tolerance

The concept of *“defect tolerance”* was proposed based on the calculated results of formation energies and transition levels for all intrinsic point defects and common defect pairs in MAPbI_3,_^[^ ^21^, ^46^ ^]^ which implies that deep defects in MAPbI_3_ invariably have high formation energy (low concentration), while defects with low formation energies form shallow levels, regardless of chemical potentials. Numerous subsequent studies^[^ ^47^, ^48^, ^49^, ^50^ ^]^ have enhanced the scope of defect tolerance by extending it to more perovskites and non‐perovskites and attempted to explain defect tolerance in LHPs using various concepts such as low‐frequency lattice phonons,^[^ ^40^ ^]^ iodine chemistry,^[^ ^51^ ^]^ and strong anharmonicity.^[^ ^52^ ^]^

Notably, defect tolerance for LHPs was discovered in 2014,^[^ ^21^ ^]^ in comparison the defect behaviors to their semiconductor counterparts such as Si, GaAs, and CdTe. It does not mean that the existence of defects in perovskites has no harmful effect at all. In the SRH recombination model,^[^ ^30^, ^31^ ^]^ shallow defects can also trap the carrier and act as scattering and recombination centers, but at a significantly lower rate than that of deep defects (see discussions in the section of *Recombination rate*). Meanwhile, deep defects also exist at relatively low concentrations yet possibly segregate into the surface/interface to act as effective recombination centers.

Apart from the dominating shallow levels of single‐point defects, a minimum of two other scenarios for defect tolerance exist:

1. A large amount of intrinsic (structural) defects exists in the system, but the defects with the opposite charge form compensating neutral defect pairs that are not harmful to carrier transport. This scenario is applicable for CuInSe_2_,^[^ ^53^, ^54^ ^]^ a prototype chalcopyrite semiconductor. More specifically, in CuInSe_2_, the individual defect of Cu vacancy ($V_{\mathrm{Cu}}^{-}$) and substitution of Cu by In (${\mathrm{In}}_{\mathrm{Cu}}^{2+}$) both have low formation energy (high concentration) and deep defect levels. According to the traditional definition of “defect tolerance” for perovskites, CuInSe_2_ should be thought to have no or weak defect tolerance. But experimental studies report a surprisingly strong electrical tolerance of CuInSe_2_ to its huge (≈1%) concentration of native defects. This obvious conflict has been solved by the formation of the neutral defect pair (2$V_{\mathrm{Cu}}^{-}$+${\mathrm{In}}_{\mathrm{Cu}}^{2+}$), which has an unusually low formation energy and strong stability, due both to the relative ease of generating Cu vacancies and to the attractive interactions between $V_{\mathrm{Cu}}^{-}$ and ${\mathrm{In}}_{\mathrm{Cu}}^{2+}$. More interestingly, all deep defect levels of V_Cu_ and In_Cu_ (which originally act as the recombination centers) are removed from the band gap during the pairing process, indicating the electrically inactive nature for the defect pair (2$V_{\mathrm{Cu}}^{-}$+${\mathrm{In}}_{\mathrm{Cu}}^{2+}$). This finding reminds us that the electrical behaviors and formation energies related to not only the single point defects but also the possible formation of defect pairs through charge compensation should be checked when rigorously assessing the defect tolerance of semiconductors. In fact, perovskites are also partially prone to such defect compensation.^[^ ^55^, ^56^, ^57^ ^]^ Hong et al.^[^ ^55^ ^]^ reported that regardless of the neutral Schottky defects, such as PbI_2_ and MAI vacancy, and Frenkel defects associated with Pb, I, and MA vacancies, such defects would not reach deep levels, despite some defects possessing low formation energies. Walsh et al.^[^ ^57^ ^]^ reported similar results: charged point defects in LHP_S_ could be compensated by the formation of Schottky defects, including PbI_2_ and MAI vacancies, which were predicted to have concentrations exceeding 0.4% and lead to shallow levels.
2. Despite the presence of deep defects in the system, they are not effective recombination centers because of phonon blockage, for example.^[^ ^40^ ^]^ It was thought that the photogenerated carriers were only coupled with low‐frequency phonons in MAPbI_3_ and electron and hole states overlapped weakly, which two factors appreciably decreased the nonadiabatic coupling, making charge recombination in MAPbI_3_ not enhanced, regardless of whether the defects introduce a shallow or deep band state. However, their calculations are challenging.^[^ ^58^ ^]^

In this sense, for a more precise definition, defect tolerance can be defined as the formed defects in the material that does not limit the carrier transport. This definition leads to new strategies for designing materials with defect tolerance. In conventional wisdom, the antibonding nature of the valence band maximum and ionicity of the conduction band minimum were considered as the sources of defect tolerance because they did not facilitate the formation of deep defect levels. However, according to new scenarios, deep levels may not matter as long as it can either be compensated or exist in a soft lattice. This paves a new way for designing materials with defect tolerance, which deserve further investigation.

## 4 Defects in Soft‐Lattice Crystal

A unique feature of halide perovskite is its soft lattice. Unlike traditional semiconductor solar cells,^[^ ^59^ ^]^ such as Si (bulk modulus: 98 GPa), GaAs (75 GPa), and CdTe (42 GPa), the lattice of LHPs is extremely soft with a bulk modulus of less than 14 GPa.^[^ ^19^ ^]^ This soft lattice results in at least three crucial facts, that is, the dynamical lattice, ease of lattice expansion/compression, and defect diffusion, which play crucial roles on defect properties.

### 4.1 Dynamical Lattice

At room temperature, the crystal lattice vibrates around the perfect lattice sites with an amplitude of ≈0.3 Å. The vibration models are well described in phonon theory under harmonic approximation.^[^ ^34^, ^60^ ^]^ Molecular dynamics (MD) simulations show that the atomic vibrations in halide perovskites are considerably stronger at room temperature with amplitudes of ≈0.6 and 1.2 Å for Pb and I, respectively.^[^ ^19^, ^20^ ^]^ This large atomic fluctuation in line with the intrinsic double‐well potential leads to strong anharmonicity in halide perovskites.^[^ ^61^ ^]^ In static defect calculations [cf. Equations ((1), (2), (3), (4), (5), (6), (7))], the atomic structures are fully relaxed, corresponding to the state at 0 K. This method may be applicable for traditional semiconductors with stiff lattices because of weak temperature‐activated structural vibrations and impact on defect behaviors at room temperature.^[^ ^19^, ^20^ ^]^ For materials with soft lattices such as LHPs, strong lattice fluctuation causes significant fluctuations of defect configurations, thus leading to significantly different defect properties with respect to static calculations. These temperature‐dependent defect properties are recently noted by Sun et al.^[^ ^62^ ^]^ and Huang et al.^[^ ^63^ ^]^ Sun et al. noticed that the total energy of the defective supercell for α‐CsPbI_3_ was randomly affected by structural distortions located away from the defect and then proposed an approach based on MD to get an ensemble average for successfully obtaining converged defect formation energy. Huang et al. established two fundamental rules for the temperature dependence of charge‐state transition level in semiconductors and unexpectedly found that the temperature dependence of charge‐state transition level for different defects were diverse: the transition level may shallower or deeper or remain unchanged.

In addition to formation energies and transition levels, soft lattice may lead to anomalous carrier recombination processes. Chu et al. reported that the defect levels associated with I_i_ and Cs_I_ (Cs substituted by I) in β‐CsPbI_3_ showed an oscillatory behavior between shallow and deep states when MD simulations were performed at 300 K.^[^ ^64^ ^]^ Under these circumstances, the deep level of I_i_ and Cs_I_ could not act as effective carrier recombination center due to phonon blockage.

### 4.2 Lattice Expansion and Compression

The lattice of LHPs was observed to be easily expanded and compressed by applying strain engineering,^[^ ^65^ ^]^ light illumination,^[^ ^66^ ^]^ and thermal effect.^[^ ^67^ ^]^ Interestingly, some studies have reported that the stretched lattice produces the bonus for PCE improvement.^[^ ^66^ ^]^ However, other studies have proposed that the compressed lattice benefits the prolongation of carrier lifetimes;^[^ ^68^, ^69^ ^]^ this apparent conflict baffles researchers. Several studies attempted to rationalize the experimental observations from the viewpoint of regulating defect properties by lattice expansion or compression,^[^ ^70^, ^71^ ^]^ based on the finding that the formation energies, defect levels, and diffusion barriers can be significantly altered when minimal strain is applied. These attempts provide researchers more insights on defect properties under the applied strain, but the conflicting explanations are confusing. Qiao et al. reported the compressed lattice eliminated the deep defect levels associated with I_i_ in MAPbI_3_ and then inhibited non‐radiative recombination.^[^ ^70^ ^]^ Zhang et al. reported that the nonradiative capture coefficient associated with I_i_, a dominant recombination center in MAPbI_3_ and FAPbI_3_, had been suppressed by one order of magnitude with 1% lattice expansion, which was responsible for the improved PCE.^[^ ^71^ ^]^ Considering that strain engineering is easily carried out for LHPs in experiments, a deep and accurate understanding of the impact of lattice compression and expansion on defect properties and the performance of PSCs is crucial for scientists to develop PSCs with improved PCE by designing appropriate strain strategies. To achieve this goal, we must clarify and solve two core issues: i) why do different experimental results show that sometimes lattice compression or lattice expansion is beneficial? ii) How can the basic consensus on the influence of lattice modification on defect properties and then carrier recombination be achieved? A possible explanation is that the dominant defects vary in different experiments and are sensitive to growth conditions, and the negative effects of various dominant defects are suppressed by lattice expansion and compression. Meanwhile, more efforts need to be focused on further checking the credibility of theoretical results.

### 4.3 Defect Diffusion

Defect migration is an interesting feature of LHPs observed in experiments and is usually considered as the reason for hysteresis in the current–voltage curve and weak stability of LHPs. Although diffusion barriers and paths have been extensively investigated,^[^ ^72^, ^73^, ^74^, ^75^ ^]^ the influence of defect migrations on carrier recombination is poorly understood. Defect diffusion is always accompanied by the breakage of bonds, resulting in dangling bonds.^[^ ^75^ ^]^ Considering that the time scale needed for defect diffusion is several orders of magnitude longer than the time scale for carrier recombination, the presence of dangling bonds during defect migration may create deep levels and then accelerate nonradiative carrier recombination. Tong et al. found that the diffusion of iodine vacancy (V_I_) can speed up nonradiative recombination by an order of magnitude.^[^ ^76^ ^]^ In addition to V_I_, the diffusion of other defects, such as iodine interstitial and lead ions, and possible defect segregation and accumulation at GBs and interfaces via diffusion deserve further investigation.

## 5 Defects at the Grain Boundary, Interface and Interphase Boundaries

Apart from point defects, defects can exist in LHPs in the form of extended defects such as GBs, surfaces, interfaces, and interphase boundaries (IBs). Experiments have shown that defect density at the surfaces/interfaces was two to four orders of magnitude higher than that in bulk crystals,^[^ ^77^ ^]^ which directs attention to defects at the surfaces/interfaces. In contrast to the large theoretical effort focused on studying point defects in LHPs, theoretical studies on the extended defects are considerably fewer. Earlier simulations on interfaces between LHPs and electron transport layer (ETL), such as LHPs/TiO_2_^[^ ^78^, ^79^ ^]^ and LHPs/SnO_2,_^[^ ^80^ ^]^ show that the electron–hole recombination across the LHP/ETL interface constitutes a major pathway of energy losses, which needs to be seriously addressed. Theoretical studies on the interface between LHPs and the hole transport layer (HTL) are fewer because of the complex structures of HTL, for example spiro‐OMeTAD.

Reliable calculations on extended defects in LHPs are challenging mainly because of their complicated structures. Earlier calculations on GBs^[^ ^81^ ^]^ adopted the structural models of ∑5(310), which was borrowed from the transmission electron microscopy (TEM) image of SrTiO_3_. The atomic resolution of GBs of LHPs by TEM is challenging because of the fragile organic molecules and soft inorganic lattice that are sensitive to high‐energy electron beam irradiation. Recently, a technological breakthrough to obtain the atomic‐scale microstructure of metal halide perovskites was achieved using low‐dose low‐angle annular dark‐field scanning TEM imaging,^[^ ^82^ ^]^ which provides new opportunities for the theoretical study of GBs and IBs in LHPs.^[^ ^83^ ^]^

The atomic structures of interfaces are more challenging because they involve two different materials. To date, no atomic resolutions on interface structures are available experimentally. In principle, one can build reasonable GB and interface structures by minimizing system energy through structural searching approaches. However, due to the complexity of the GB and interface problem, which requires numerous atoms in the simulation, a straightforward structural search based on the expensive first‐principles calculations is not feasible. Recently, Gong et al.^[^ ^84^, ^85^ ^]^ has successfully adopted machine‐learning methods for structural search and prediction, such as applying an adaptive genetic algorithm and training neutral network potentials, to resolve the atomic structures of GB in SrTiO_3_ and the CdS/CdTe interface with a significantly reduced computational cost. The presented approaches in these studies can also be transferred to solve the atomic structures of GBs and interfaces referring to LHPs.

Point defects may segregate into GBs, IBs, and interfaces, resulting in structural complexity. Defect segregation at GBs and interfaces play crucial roles in carrier transportation in conventional solar cell absorbers such as CIS,^[^ ^86^ ^]^ CIGS,^[^ ^87^ ^]^ and CZTS.^[^ ^88^ ^]^ In halide perovskites, Cl and O are assumed to be able to segregate into GBs and interfaces and heal the detrimental defects.^[^ ^20^ ^]^ Recently, considerable experimental efforts^[^ ^89^, ^90^, ^91^, ^92^, ^93^ ^]^ have been focused on passivating detrimental defects at the surface, GBs, and interfaces with various Lewis acid/base species. Qualitative claims stated that Lewis acids passivate negatively charged defects, while Lewis bases passivate positively charged defects. In our opinion, several unclear issues remain: 1) which defects at the interface are passivated by Lewis acids/bases? 2) Which functional groups of Lewis acid/base play the core role? 3) What is the atomic‐level passivation mechanism? 4) Are there any general rules for selecting optimal passivators among thousands of chemical species, thus preventing *trial‐and‐error* in experiments?

## 6 Defect‐Triggered Phase Transition and Degradation

It is known that perovskites with ABX_3_ composition can adapt different crystal structures, which relies on the size and interaction of the A cation and the corner‐sharing BX_6_ octahedra. It is widely accepted that Goldschmidt tolerance factor (*t*) is a general and reliable descriptor to predict which structure is preferred,^[^ ^94^ ^]^ with the definition as follow:

(9) $$t\;=\frac{r_{A}+r_{X}}{\sqrt{2}\left(r_{B}+r_{X}\right)}\;$$

where *r*_A_, *r*_B_, and *r*_X_ is the radius of the A‐site, B‐site cation and X‐site anion, respectively. In general, the perovskite structure (α‐, β‐, and γ‐phase) is preferred for the material with 0.8 < *t* < 1.0.^[^ ^95^ ^]^ Unfortunately, both CsPbI_3_ (*t* < 0.8) and FAPbI_3_ (*t* > 1.0) possess an inappropriate Goldschmidt tolerance factor, resulting in that the non‐perovskite structure (δ‐phase) is more stable that the perovskite structures. Therefore, CsPbI_3_ and FAPbI_3_ samples suffer from extremely weak phase stability alongside a strong tendency to transform from the black perovskite phase to the yellow non‐perovskite phase, which is photo‐inactive. Inhibiting this undesired phase transformation has become a hot topic and recently drawn intense interest.^[^ ^9^, ^96^, ^97^, ^98^, ^99^ ^]^ Despite numerous experimental indications that defects may accelerate the phase transition and degradation of LHPs, current computational studies about defects still mainly focus on its optoelectrical impact on the performance of PSCs while rarely taking phase stability into account.

**Figure 6:**
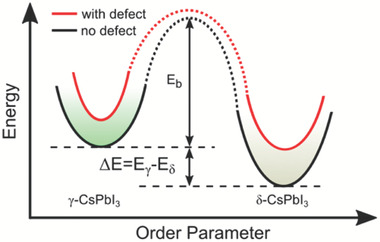
Energy diagram schematically illustrating the relative stabilities of the perovskite and non‐perovskite‐phase CsPbI_3_.

According to experiments, the passivation or suppression of intrinsic defects (e.g. cation and anion vacancies) in LHPs and the intentional introduction of special non‐intrinsic defects, such as the mixing of A sites (MA^+^, FA^+^, Cs^+^), the partial replacement of Pb^2+^ by other metal cations (e.g. Sn^2+^, Bi^3+^, La^3+^) and the substitution of I^−^ by anions including Br^−^, Cl^−^, SCN^−^, and COOH^−^ through doping, all can significantly improve phase stability.^[^ ^11^, ^96^, ^100^, ^101^, ^102^, ^103^ ^]^ These reports clearly indicate that the phase stability of LHPs is closely related to interior defects, but theoretical studies that profoundly investigate how defects impact the phase stability are rarely conducted. The current understanding of the effects of defects on stability is limited to the thermodynamic knowledge that the non‐perovskite phases of CsPbI_3_ and FAPbI_3_ have lower energies than their perovskite phase counterparts and that this energy difference fundamentally promotes transition to the undesired phase. Accordingly, calculations show that defects enhancing the energy difference lowers the phase stability of LHPs and vice versa.^[^ ^104^, ^105^ ^]^ Such argument is rational; however, important kinetic processes such as the phase transition path and barrier are disregarded (cf. **Figure** **6**).

According to the theory of chemical reaction equilibrium,^[^ ^106^, ^107^, ^108^, ^109^ ^]^ the energy difference (Δ*E*) between the initial state (IS) and final state (FS) determines the automatic reaction direction (from high‐energy state to low‐energy state), while the kinetic barrier (*E*_b_) determines the phase transition rate ν, with $\upsilon \propto \exp(\frac{-E_{b}}{k_{B}T})$. In principle, the metastable state can be stabilized to prolong its lifetime through using a high barrier to significantly lower the phase transition rate, exemplified by graphite and diamond.^[^ ^110^ ^]^ Therefore, not only energy difference (Δ*E*) between perovskite and non‐perovskite phases but also the rate‐determining transition barrier (*E*_b_) should be quantitatively calculated. Moreover, their dependence on defects should be clarified, and strategies relying on enhanced *E*_b_ by tuning defects to stabilize the perovskite phase should be rationally established.^[^ ^111^ ^]^ Computational efforts are first required to focus on clarifying the phase transition path and barrier for structural modes with and without various defects. Thereafter, the mechanism for assessing the phase stability of LHPs while simultaneously considering the contribution from Δ*E* and *E*_b_, needs to be developed. Under this scheme, the overall influence of various defects on the phase stability may be quantitatively assessed, with the ultimate aim of providing accurate and useful guidance for experimental researchers to design efficient stabilization strategies by the targeted inhibition of detrimental defects and introduction of beneficial defects.

## 7 Accelerating Defect Research via High‐Throughput Computing and Machine Learning

Current defect calculations mainly rely on DFT calculations, which are conventionally time‐consuming and expensive because they require a large supercell and elaborate correction approaches. Therefore, conventional defect calculations usually focus on only one defect or one kind of target materials. With increasing studies on LHPs, considerable data have been accumulated in both experimental and computational studies, which necessitate advanced techniques such as machine learning (ML) to establish useful rules and avoid *trail‐and‐error* attempts. Meanwhile, great efforts are focused on exploring new perovskites^[^ ^112^, ^113^, ^114^ ^]^ or materials^[^ ^115^ ^]^ beyond perovskites for solar cell absorbers. Such research usually requires high‐throughput computing to screen optimal data among thousands of potential candidates. Existing studies^[^ ^116^, ^117^, ^118^, ^119^ ^]^ are mainly focused on bulk properties of candidates, such as formation energy, bandgap, and effective mass. Because defects are crucial, high‐throughput computing and machine learning for defect studies are necessary. A few recent studies have started to extend the application of ML to impurity level prediction in Cd‐based chalcogenides,^[^ ^120^ ^]^ oxygen vacancies in oxide perovskites,^[^ ^121^, ^122^ ^]^ and even the slow evolution of complex atomic and electronic structures of grain boundaries in perovskites,^[^ ^123^ ^]^ implying promising applications of ML for defect study in LHPs from the theoretical calculation perspective.

**Figure 7:**
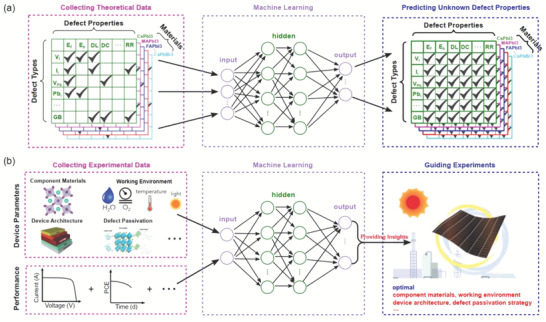
Schematic illustration of the machine learning for a) fast predicting defect properties based on the data from existing theoretical data, and b) finding the hidden rule correlating device parameters and performance and then guiding experiment for optimal parameters to achieve high‐efficiency stable PSCs.

Considering that there are still a large number of unknown defect properties in PSCs, which may be limited by the calculation approaches or resources, it is suggested researchers can collect the abundantly existing theoretical data to serve as the input for the training of ML models, and then use the well‐trained models to find the underlying rules for defects in LHPs, thus predicting the unknown/unstudied defect properties in a fast way (cf. **Figure** **7**a).

Besides providing deeper insights and unknown information, ML could also be directly applied to guide and promote experiments, for example, screening optimal additives to passivate detrimental defects based on experimental data in PSCs. Numerous experiments have reported different additives selected for surface defect passivation and the corresponding PCE, as well as the replacement of Pb^2+^ by other metal cations and the improved lifetime of LHPs in the ambient environment. One may apply ML techniques to deal with these data and reveal the relationship between the passivation additive and PCE, as well as that between the B‐site doping atom and lifetime. In principle, we are able to collect enough experimental data concerning device parameters (e.g. absorption materials, working conditions, device architecture, defect passivation strategies, …) and performances (e.g. stability, efficiency, open‐circuit voltage, …) as the input for Machine learning, which can help us build the correlation model and then predict the best device parameters for the desired performances of PSCs (cf. Figure 7b). The proposed research plan appears to be clear and feasible. However, we should also realize the potential difficulties, such as the methods for normalizing the data reported by different groups and the selection of the appropriate descriptor. In addition, an open‐access database collected from more than 42 400 photovoltaic devices with up to 100 parameters per device has been built,^[^ ^124^ ^]^ this database can provide abundant data for ML exploration based on experimental results. Overall, we firmly believe that ML can play an important role in helping researchers design the best passivation and doping strategy by defect engineering to improve the performance of PSCs.

## 8 Conclusion

Owing to the consistent contribution in the last 30 years, computation is becoming an indispensable route to understanding defects in solids and has recently been widely used in investigating perovskite solar cells. In this Perspective, we considered a brief review of the current knowledge concerning computational studies on defects in LHPs to present the unsolved problems and proposed possible research directions in the future. In our opinion, three aspects deserve further consideration and investigation. The first is developing advanced approaches for deeply understanding defects, such as the temperature‐dependent effect on defects and their impact on carrier transportation and recombination. The second is initiating data‐driven defect research to meet the requirements for rapidly screening functional passivators and materials. The third is establishing the link and bridge between computational studies and experimental observations, making computational studies better guide and promote experiments.

## Conflict of Interest

The authors declare no conflict of interest.
